# Inhibition of Survivin Influences the Biological Activities of Canine Histiocytic Sarcoma Cell Lines

**DOI:** 10.1371/journal.pone.0079810

**Published:** 2013-11-15

**Authors:** Hiroki Yamazaki, Satoshi Takagi, Yuki Hoshino, Kenji Hosoya, Masahiro Okumura

**Affiliations:** 1 Laboratory of Veterinary Surgery, Department of Veterinary Clinical Sciences, Graduate School of Veterinary Medicine, Hokkaido University, Hokkaido, Japan; 2 Veterinary Teaching Hospital, Graduate School of Veterinary Medicine, Hokkaido University, Hokkaido, Japan; Philipps University, Germany

## Abstract

Canine histiocytic sarcoma (CHS) is an aggressive malignant neoplasm that originates from histiocytic lineage cells, including dendritic cells and macrophages, and is characterized by progressive local infiltration and a very high metastatic potential. Survivin is as an apoptotic inhibitory factor that has major functions in cell proliferation, including inhibition of apoptosis and regulation of cell division, and is expressed in most types of human and canine malignant neoplasms, including melanoma and osteosarcoma. To investigate whether survivin was expressed at high levels in CHS and whether its expression was correlated with the aggressive biological behavior of CHS, we assessed relation between survivin expression and CHS progression, as well as the effects of survivin inhibition on the biological activities of CHS cells. We comparatively analyzed the expression of 6 selected anti-apoptotic genes, including survivin, in specimens from 30 dogs with histiocytic sarcoma and performed annexin V staining to evaluate apoptosis, methylthiazole tetrazolium assays to assess cell viability and chemosensitivity, and latex bead assays to measure changes in phagocytic activities in 4 CHS cell lines and normal canine fibroblasts transfected with survivin siRNA. Survivin gene expression levels in 30 specimens were significantly higher than those of the other 6 genes. After transfection with survivin siRNA, apoptosis, cell growth inhibition, enhanced chemosensitivity, and weakened phagocytic activities were observed in all CHS cell lines. In contrast, normal canine fibroblasts were not significantly affected by survivin knockdown. These results suggested that survivin expression may mediate the aggressive biological activities of CHS and that survivin may be an effective therapeutic target for the treatment of CHS.

## Introduction

Canine histiocytic sarcoma (CHS) is an aggressive malignant neoplasm originating from histiocytic lineage cells, including dendritic cells (DCs) and macrophages, and is characterized by progressive local infiltration and a very high metastatic potential [1], [2]. Monotherapy with lomustine (CCNU) and multidrug therapy with various combinations of prednisone, doxorubicin (DOX), CCNU, and other alkylating agents are commonly used for the treatment of CHS. However, CHS often acquires early multidrug resistance to these antitumor agents, leading to a median survival time of less than 100 days [3], [4]. Hemophagocytic histiocytic sarcoma originating from macrophages shows aggressive hemophagocytic activity in addition to the common progressive pathological mechanism and chemoresistance [5], [6], resulting in a relatively poor prognosis compared to CHS originating from DCs. Therefore, it is necessary to identify endogenous factors that are related to these aggressive behaviors and to subsequently develop more effective therapies against CHS.

Survivin belongs to the inhibitor of apoptosis (IAP) family and is known to be an anti-apoptotic factor [7]. Unlike other IAP family members, survivin has 2 major functions in cell proliferation: inhibition of apoptosis and regulation of cell division [7], [8]. Survivin is highly expressed in most types of human and canine malignant neoplasms, including melanoma and osteosarcoma [9]–[13], but is expressed at very low levels in normal cells and benign tumors, with the exception of hematopoietic progenitor cells and fibroblasts [14]–[18]. Some studies have shown that increased expression of survivin promotes chemoresistance and proliferation in human cancer cells [10], [11]. Moreover, survivin expression has been shown to correlate with aggressive behavior and prognosis in malignant neoplasms, including canine osteosarcoma [13], [19], [20], and has been proposed as an effective therapeutic target for canine osteosarcoma [13]. The expression of survivin also correlates with the progression of some pathological processes of cancer, functioning through apoptotic mechanisms different from those of other IAP and Bcl-2 family members [8], and is expected to be a potential target for human and canine antitumor therapy [12], [13].

Small interfering RNA (siRNA), a method of RNA interference (RNAi), is frequently used as a valuable tool to inhibit the expression of target genes and is a common method for basic studies of molecular targeted therapy [13], [21]–[23]. This method of RNAi involves post-transcriptional gene silencing via a process in which double-stranded RNA inhibits gene expression in a sequence-dependent manner through degradation of the corresponding mRNA [22], [23]. Such RNAi-mediated knockdown of gene expression has been successfully observed in human and canine cells cultured in vitro [21], [13], and inhibition of survivin expression has been achieved using this technology [21]–[23].

Based on this background, we hypothesized that survivin was specifically expressed at high levels in CHS and that enhanced survivin expression would correlate with the aggressive behavior of CHS. To verify this hypothesis would require demonstration that survivin is specifically expressed in CHS at high levels compared to other anti-apoptotic factors and that survivin expression is correlated with the biological activities of CHS cells. The aims of this study were to comparatively analyze the gene expression levels of survivin, in addition to IAP and Bcl-2 family members, in dogs with histiocytic sarcoma (hereafter referred to as HS dogs) and to evaluate the effects of survivin knockdown using siRNA on biological activities, including cell viability, chemosensitivity and hemophagocytic activity, in 4 CHS cell lines derived from different sources.

## Materials and Methods

### Specimen collection and handling

The entire procedure of animal use in this study was approved by Institutional Animal Care and Use Committee, the serial number of approval as #1120, Graduate School of Veterinary Medicine, Hokkaido University, which has been fully accredited by the Association for Assessment and Accreditation of Laboratory Animal Care International. Specimens were collected from 30 HS dogs by excisional or needle biopsy at initial medical examinations in a veterinary teaching hospital at Hokkaido University between October 2009 and September 2012. All specimens were confirmed by histopathological examination. Samples of HS dogs were collected by excisional biopsy or needle core biopsy. For histopathological evaluation, one block of each specimen was immersed in 10% formalin solution at room temperature or was freshly frozen in liquid nitrogen, then stored at –80°C. Simultaneously, for extraction of mRNA, another block from each sample was immersed in RNA preservative solution (RNAlater; Ambion, Grand Island, NY, USA) for 24 h at 4°C, and then stored at –80°C until use. For definitive diagnosis, immunohistochemical staining using anti-MHC class II and anti-CD18 antibodies, and histopathological evaluation by hematoxylin-eosin staining was performed by two specialized pathologists in different two commercial laboratories.

### Real-time PCR

Bcl-2 family members, including Bcl-2, Bcl-xL, and Mcl-1, and IAP family members, including cIAP-1, cIAP-2, XIAP, and survivin, were selected as anti-apoptotic factors for this study. Expression of these mRNAs in specimens from 30 HS dogs were evaluated using real-time reverse transcription-polymerase chain reaction (qRT-PCR). Trizol (Invitrogen Life Technologies, Carlsbad, CA, USA) was used for isolation of total RNA, and specimens were treated with DNase (Recombinant DNase I; Takara Bio, Inc., Shiga, Japan) to remove contaminating DNA. The integrity of the isolated RNA was determined by agarose gel electrophoresis, which allowed for differentiation between degraded (smear) and intact RNA (ratio 28S/18S rRNA ribosomal band 2:1), and the quantity of isolated RNA was measured by spectrophotometer at A260/A280 (ratio had to be between 1.8 and 2.0). Reverse transcription for cDNA synthesis was performed using an Oligo dT primer and Moloney Murine Leukemia Virus reverse transcriptase Kit (M-MLV RT Kit; Invitrogen Life Technologies), and all of synthesized cDNAs were adjusted to a concentration of 20 µg/mL. Candidate internal reference genes were selected for quantitative analysis, including ACTB (actin, beta), GAPDH (glyceraldehyde-3-phosphate dehydrogenase), HPRT (hypoxanthine phosphoribosyltransferase 1), RPL13A (ribosomal protein L13a) and TBP (TATA box-binding protein) [27], [28]. For normalization of the amount of cDNA sample, expressions of these 5 genes were quantified, and accuracies as internal control were analyzed using geNorm (version 3.5 software) [28], [29]. Hypoxanthine phosphoribosyltransferase 1 gene was determined as an internal reference from the result of this analysis. All target primer sequences used in this study were designed using the Primer3 interface (http://frodo.wi.mit.edu/) or previous reports (Table 1) [13], [28], [30], [31]. Real-time PCR was performed using Rotor-Gene Q (Qiagen, Hilden, Germany) with KAPA SYBR FAST qPCR Master Mix (KAPA Biosystems, Boston, MA, USA) according to the manufacturer’s instructions. cDNA samples were subjected to activation at 95°C for 3 min, then 40 cycles of denaturation at 95°C for 20 s, and annealing/extension at 60°C for 20 s. The relative quantification in gene expression was determined using the 2-ΔΔCt method. All mRNA expression levels were normalized to those of canine fibroblasts and are represented as relative quantitative values, which were divided by expression levels in fibroblasts. Data were expressed as mean values of experiments performed in triplicate. Sequence analysis was performed by query for GenBank with the basic local alignment search tool algorithm (BLAST; http://blast.ncbi.nlm.nih.gov) [32]. In addition, all DNA fragments were extracted from the gel using Quantum Prep Freeze 'N Squeeze DNA gel extraction spin columns (Bio-Rad Laboratories, Hercules, CA, USA), and then subjected to Hokkaido System Science for DNA sequencing. Specificities of all PCR amplicons were confirmed by melt curve analysis.

**Table 1:** Target primer sequences used in this study.

Target genes	Sequences	Amplicon size (bp)	Genbank accession #
HPRT	Forward 5′-TGCTCGAGATGTGATGAAGG-3′	192	NM001003357
	Reverse 5′-TCCCCTGTTGACTGGTCATT-3′		
Bcl-2	Forward 5′-TGGAGAGGGTCAACCGGGAGATG-3′	87	AB154172
	Reverse 5′-AGGTGTGCAGATGCCGGTTCAGG-3′		
Bcl-xL	Forward 5′-GCCTTTTTCTCCTTGGGTGG-3′	185	AB073983
	Reverse 5′-CTCTCGGCTGCTGCATTGTT-3′		
Mcl-1	Forward 5′-TGTGGCCAAACACTTGAAGA-3′	138	AB093582
	Reverse 5′-GTCAAACAAAGAGGCTGGGA-3′		
cIAP1	Forward 5′-AGGCGTCCCCGTGTGCGAGAG-3′	96	XM858260
	Reverse 5′-TAGCATCAGGCCGCAGCAGAAGC-3′		
cIAP2	Forward 5′-AGGCCAATGTAATTAATAAACAGGA-3′	95	DQ223014
	Reverse 5′-AACTAAGACAGTATCAATCAGTTCTCTC-3′		
Survivn	Forward 5′-TCGAAGAGACCGCAAAGAAAGTGC-3′	181	AY741504
	Reverse 5′-GAATTGTGGCCGTTCTCCTTTCCT-3′		
XIAP	Forward 5′-ACTATGTATCACTTGAGGCTCTG-3′	80	AY603038
	Reverse 5′-AGTCTGGCTTGATTCATCTTGTG-3′		
MGMT	Forward 5′-GATGAGGAGCAATCCTGTGC-3′	88	NM001003376
	Reverse 5′-GAGTCCTCCGGTGTAGTTGC-3′		
ABCB1	Forward 5′-ACTCGGGAGCAGAAGTTTGA-3′	95	NM001003215
	Reverse 5′-AATGAGACCCCGAAGATGTG-3′		
ABCC2	Forward 5′-GAGCTGGCTCACCTCAAAAC-3′	103	NM001003081
	Reverse 5′-GTAGCTGCCTCTGCCCTATG-3′		

### Cell lines and culture

Four CHS cell lines (CHS-4, CTT, DH82, and LHS) and canine fibroblasts (control cells) were used in this study. CHS-4 [24] cells were kindly provided by Dr. Bonkobara, Department of Veterinary Clinical Pathology, Nihon Veterinary and Life Science University, and CTT [25] cells were provided by Dr. Maruo, Department of Veterinary Medicine, Gifu University, respectively. DH82 cells were purchased from DS Pharma Biomedical (Osaka, Japan). LHS cells and fibroblasts were established in this study as described below. All cell lines were cultured and maintained in Dulbecco’s modified Eagle’s medium (DMEM; Gibco-BRL, Paisley, UK) supplemented with 10% heat-inactivated fetal bovine serum (FBS; Biomedical Inc., Aurora, OH, USA) and antibiotics (100 IU/mL penicillin and 100 µg/mL streptomycin; Wako Pure Chemical Industries, Ltd., Osaka, Japan) in a humidified incubator with 5% CO_2_ at 37°C.

LHS cells were freshly isolated from a 12-year-old male, castrated Welsh Corgi with primary lung HS using a previously reported procedure [24]. Briefly, tumor tissue collected from the dog was finely minced and cultured using the medium and culture conditions described above and serially passaged by trypsinization. Adherent cells were successfully established after over 50 passages without further additives. Tumor cells (1×10^6^ cells) were subcutaneously injected into three 6-week-old male severe combined immunodeficiency disease (SCID) mice (C.B-17/lcr-scid/scid Jcl; CLEA Japan, Inc., Tokyo, Japan). Solid masses (approximately 1,000 mm^3^) were produced at the injection site at 4 weeks after injection. These tumor tissues were collected from the mice and identified as original CHS as described below. Morphological and cytochemical findings were positive for α-naphthyl butyrate esterase (Kit No. 181-B; Sigma-Aldrich, St. Louis, MO, USA), and immunocytochemical staining was positive for vimentin (clone Vim3B4; Dako, Glostrup, Denmark) and lysozyme (Dako), but negative for cytokeratin (clone AE1/AE3; Dako).

Fibroblasts were freshly isolated from the abdominal subcutis of a healthy 1-year-old, intact female beagle using a previously reported procedure [26]. Briefly, collected tissues were finely minced and incubated in serum-free DMEM supplemented with 4 mg/mL collagenase type IA (Wako) at 37°C in 5% CO2 for 4 h. Then, cells were suspended in DMEM with 10% FBS and antibiotics under the same conditions to obtain monolayer adherent cells. Cells between the second and fifth passages were used for this study. Cell viability always exceeded 90% by the trypan blue exclusion test.

### Reagents

siRNA – A custom siRNA targeting canine survivin (target sequence: 5′-CAAGCAGAAAGAATTCGAA-3′, 3′-overhang dTdT [sense/antisense], 27 bp) and a scrambled siRNA as a negative control (target sequence: 5′-ATCCGCGCGATAGTACGTA-3′, 3′-overhang dTdT [sense/antisense]) were designed as previously reported [13], [21] and purchased from Cosmo Bio Co., Ltd. (Tokyo, Japan). Transfection with siRNA was accomplished using cationic liposome (LipoTrust™ *EX* Oligo; Hokkaido System Science, Hokkaido, Japan), according to the manufacturer’s instructions. CHS cells and canine fibroblasts were seeded with DMEM supplemented with 10% FBS in 96-well plates (Costar Corning Inc., Corning, NY, USA) at 1–2×10^4^ cells/well or 6-well plates (Costar Corning Inc.,) at 1–2×10^5^ cells/well and incubated overnight in an incubator with 5% CO_2_ at 37°C. Survivin siRNA and scrambled siRNA were diluted in deionized distilled water (DDW) according to the manufacturer’s instructions. Diluted siRNAs were complexed in 0.5 µL of cationic liposome dissolved in 1 mL DDW for 96-well plates (10 µL of cationic liposome for 6-well plates) and were incubated at room temperature for 20 min. Then, 0.5 µL of siRNA/liposome complexes were added to each well for 96-well plates (10 µL for 6-well plates), and cells were incubated in an incubator with 5% CO_2_ at 37°C.

### Survivin expression

Cell lines were treated with siRNA in 60-mm cell culture dishes (Costar Corning Inc.,). Cells were harvested at 12, 24, 48, and 72 h after transfection with siRNA, and expression of survivin mRNA was evaluated by qRT-PCR as described above. In addition, At 48 h after transfection with siRNA, survivin protein in cells evaluated by western blotting as follows. cells were washed twice ice-cold Tris-buffered saline (TBS, pH 7.4; Sigma-Aldrich) and lysed with 100 µL of RIPA buffer (Sigma-Aldrich). The protein concentration of each lysate was determined by Bradford protein assay using bovine serum albumin (BSA; Sigma-Aldrich), and 30 µg of protein was loaded for each sample. Proteins were denatured, subjected to SDS-PAGE using 10% polyacrylamide gels (Wako), and electrotransferred into nitrocellulose membranes (Whatman, Dassel, Germany). Membranes were blotted with blocking solution containing 10 mM Tris-HCl, 0.15 M NaCl (Wako), 0.1% Tween-20 (Pharmacia Biotech, Uppsala, Sweden), and 1% BSA (Sigma-Aldrich) in TBS with 0.05% NaN_3_ for 1 h at room temperature. Membranes were incubated overnight at 4°C with primary antibodies against β-actin (ab8227; Abcam, Cambridge, MA, USA) and survivin (clone NB500-201; Novus Biologicals, Littleton, CO, USA) at a dilution of 1∶1,000 in blocking solution containing 5% BSA, 10 mM Tris-HCl (pH 7.4), 0.15 M NaCl, and 0.1% Tween-20. Membranes were washed 3 times for 5 min each with Tween-TBS containing 10 mM Tris-HCl (pH 7.4), 0.15 M NaCl, and 0.1% Tween-20 and incubated with anti-rabbit alkaline phosphatase conjugate IgG (Sigma-Aldrich) at a dilution of 1∶1,000 in Tween-TBS. The NBT/BCIP system (Roche Applied Science, Mannheim, Germany) was used for visualization.

### Assessment of apoptosis and cell viability

Apoptosis was evaluated using annexin V staining (Annexin V-Biotin Apoptosis Detection Kit; Biovision, Mountain View, CA, USA). CHS cells and canine fibroblasts were directly incubated on glass slides in 6-well plates and transfected with siRNA. At 48 h after transfection with siRNA, cells on the slide were rinsed with 200 µL of Binding buffer (Biovision). Then, 5 µL of Annexin V-biotin and 5 µL of propidium iodide (Biovision) were added to cells, and the cells were further incubated at room temperature for 10 min in the dark. Cells were washed once in 200 µL of Binding buffer, fixed with 2% formaldehyde in PBS for 15 min, and then washed once with PBS. One hundred microliters of PBS containing 1 mg/mL BSA was then added, and cells were washed once with PBS. Then, cells were incubated with 5 µg/mL avidin-fluorescein (streptavidin-FITC; Blue Heron Biotechnology, Inc., Bothell, WA, USA) and incubated for additional 15 min. Cytological morphology of nuclei and membranes was observed using a fluorescence microscope (BIOREVO BZ-9000; Keyence Corp., Osaka, Japan) and light microscope by Wright-Giemsa staining. For Wright-Giemsa staining, cells on glass slides were fixed with 100% methanol for 5 min and then stained with Wright-Giemsa stain modified (Sigma Diagnostics, St Louis, MO, USA) according to the manufacturer’s recommendations.

Cell viability was evaluated by methylthiazole tetrazolium (MTT) assay. CHS cells and canine fibroblasts were seeded in 96-well plates. Cells were then left untreated (control) or were transfected with scrambled siRNA or survivin siRNA. At 24 and 48 h after transfection with siRNA, 10 µL of MTT solution (10 mg/mL; Wako) was added to each well, and cells were incubated in an incubator with 5% CO^2^ at 37°C for 4 h. The supernatants were removed, and 100 µL of solution buffer (47.5 µL of deionized distilled water with 47.5 µL of N-N-dimethyl formamide and 20 mg of sodium dodecyl sulfate, pH 4.7) was added to each well. Cells were then shaken for 1 min. The absorbance of each well was measured at a wavelength of 570 nm using a Multiskan EX microplate spectrophotometer (Thermo Scientific, Waltham, MA, USA). Results were presented as the average of triplicate samples, and the experiment was repeated 3 times.

### Chemosensitivity test and expression of chemoresistnce-related genes

Chemosensitivity was evaluated by MTT assay. Cells were seeded in 96-well plates and left untreated or transfected as described above. At 48 h after transfection with siRNA, cells were treated with different concentrations (0.1–1,000 µM) of CCNU (Wako) or DOX (Wako). Twenty-four hours later, the 50% inhibitory concentration (IC50) was calculated by MTT assay as described above.

Gene expression of ATP-binding cassette transporter B1 (ABCB1) as a p-glycoprotein, ATP-binding cassette transporter C2 (ABCC2) as a multidrug-resistance protein and O6-methylguanine–DNA methyltransferase (MGMT), were analyzed using qRT-PCR. These were used for this presented study as chemoresistance-ralated genes, which are related to the acquisition of CCNU- and DOX-resistance. Cell lines were seeded in 6-well plates and left untreated or transfected with scrambled or survivin siRNAs as described above. At 48 h after transfection with siRNA, mRNAs expression were evaluated. Analysis procedure was performed by the above mentioned protocol, and target primer sequences for ABCB1, ABCC2 and MGMT were designed according to previous reports (Table 1) [28]. All mRNA expression levels were normalized to those of the same gene in untreated CHS cells (control) and are represented as the relative expression (% of control). Sequence analysis was performed as mentioned above.

### Protein expression and function assessment of ABCB1

At 48 h after transfection with siRNA, protein expression of ABCB1 in CHS cells was evaluated by western blotting using antibodies targeting ABCB1 (C219; Abcam, Cambridge, MA, USA) at a 1:500 dilution as described above. ABCB1 function (P-glycoprotein pump activity) was simultaneously assessed by staining with Hoechst-33342 using fluorescence imaging, as previously described [26], [33]. In brief, at 48 h after transfection with siRNA, the cells were incubated with 5 µg/mL of Hoechst 33342 (Hoechst dye 33342; Sigma-Aldrich) at 37°C for 90 min. After washing with cold PBS twice, nuclear morphology and cell membrane were visualized using a fluorescence microscope (BIOREVO BZ-9000; Keyence Corp).

### Phagocytic activity assay

Phagocytic activity was evaluated using latex beads (Fluoresbrite Carboxylate Microspheres [2.5% Solids-Latex] 2-µm YG; Polysciences Inc., Warrington, PA, USA) as previously described [24]. Cells were directly incubated on glass slides in 6-well plates and were transfected with siRNA. At 48 h after transfection with siRNA, latex beads (100 µL) were added each well. After 2 h, the slides were washed 3 times in PBS for removal of the extra latex beads that had not been phagocytosed. Then, slides were fixed in methanol for 15 s and stained with Wright-Giemsa staining solution (Wako). Cytological morphology was observed by light microscopy, and analysis of the phagocytic rate was performed using an image analyzer (BIOREVO BZ-9000; Keyence Corp). The total number of latex beads phagocytosed in 2,000 randomly selected viable cells was measured. Phagocytic cells were shown to be alive using trypan blue exclusion test.

### Statistical analysis

Data were expressed as the mean ± standard deviation. Dunnett’s test or one-way ANOVA followed by the Tukey post-hoc test for data with multiple comparisons was performed using commercial statistical software (IBM SPSS Statistics; SPSS Inc., Chicago, IL, USA) to determine significant differences between groups, and *P*-values of less than 0.05 were considered significant.

## Results

### Comparative analysis of Bcl-2 and IAP family gene expression in HS dogs

**Figure 1:**
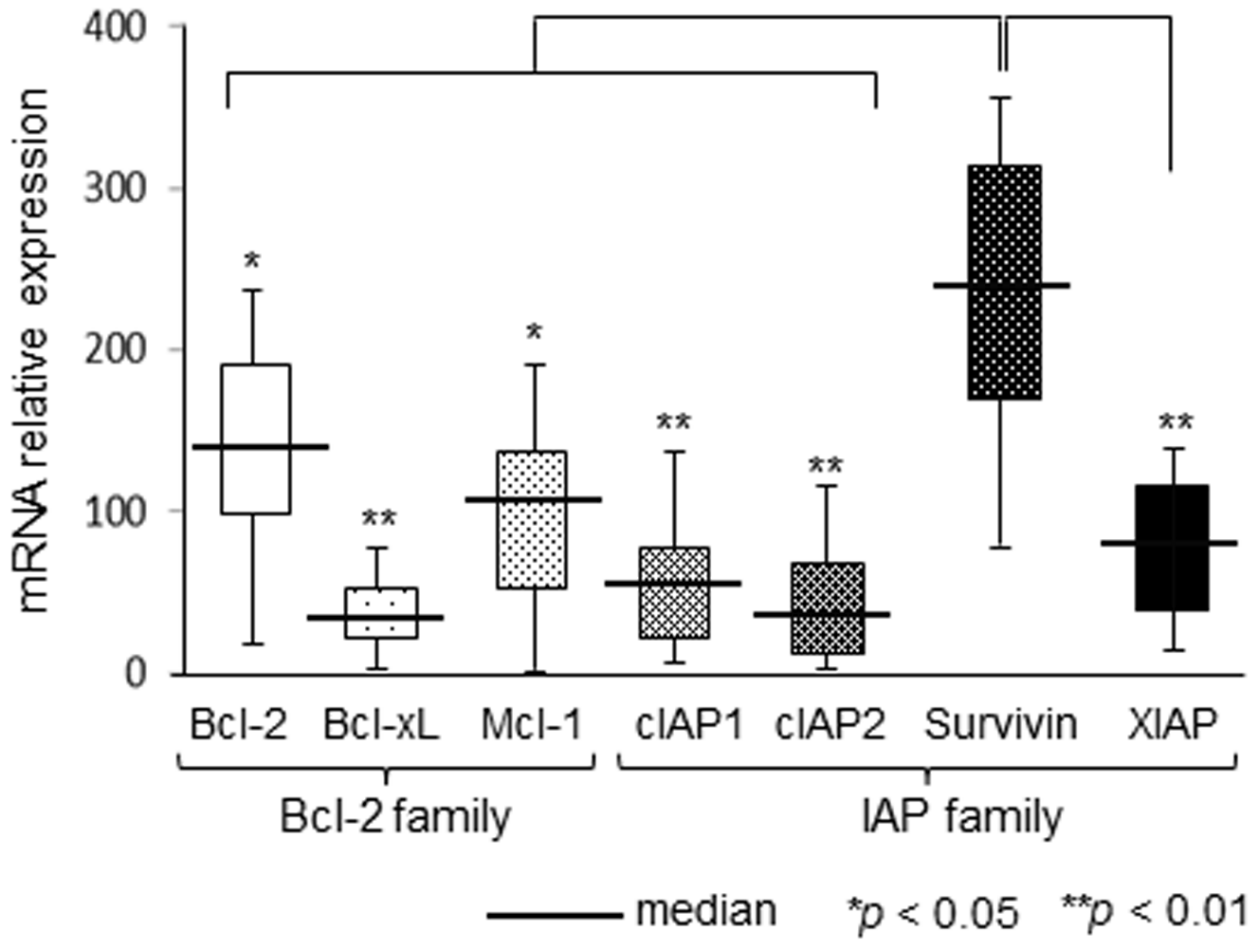
Gene expression of Bcl-2 and IAP family in 30 dogs with histiocytic sarcoma. mRNA expression levels of Bcl-2 and IAP family members in samples collected from 30 dogs with histiocytic sarcoma were analyzed using real-time reverse transcription-polymerase chain reaction (qRT-PCR). Expression levels of each gene were normalized to those of the same gene in canine fibroblasts. Each bar represents the mean ± SE from 3 separate experiments. *p<0.05 and **p<0.01 for survivin vs. the other anti-apoptotic genes (Dunnett’s test).

The expression levels of anti-apoptotic genes, including Bcl-2 family members (Bcl-2, Bcl-xL, and mcl-1) and IAP family members (cIAP-1, cIAP-2, survivin, and XIAP) in specimens collected from 30 HS dogs were comparatively analyzed using qRT-PCR (Fig. 1). The expression of survivin mRNA (median: 248, range: 80–360) in these specimens was significantly higher than those of Bcl-2 family proteins and other IAP family proteins. Additionally, the expression of Bcl-xL mRNA was the lowest of the anti-apoptotic genes, with the median Bcl-xL mRNA expression level approximately one-tenth that of survivin.

### Survivin expression in CHS cell lines

**Figure 2:**
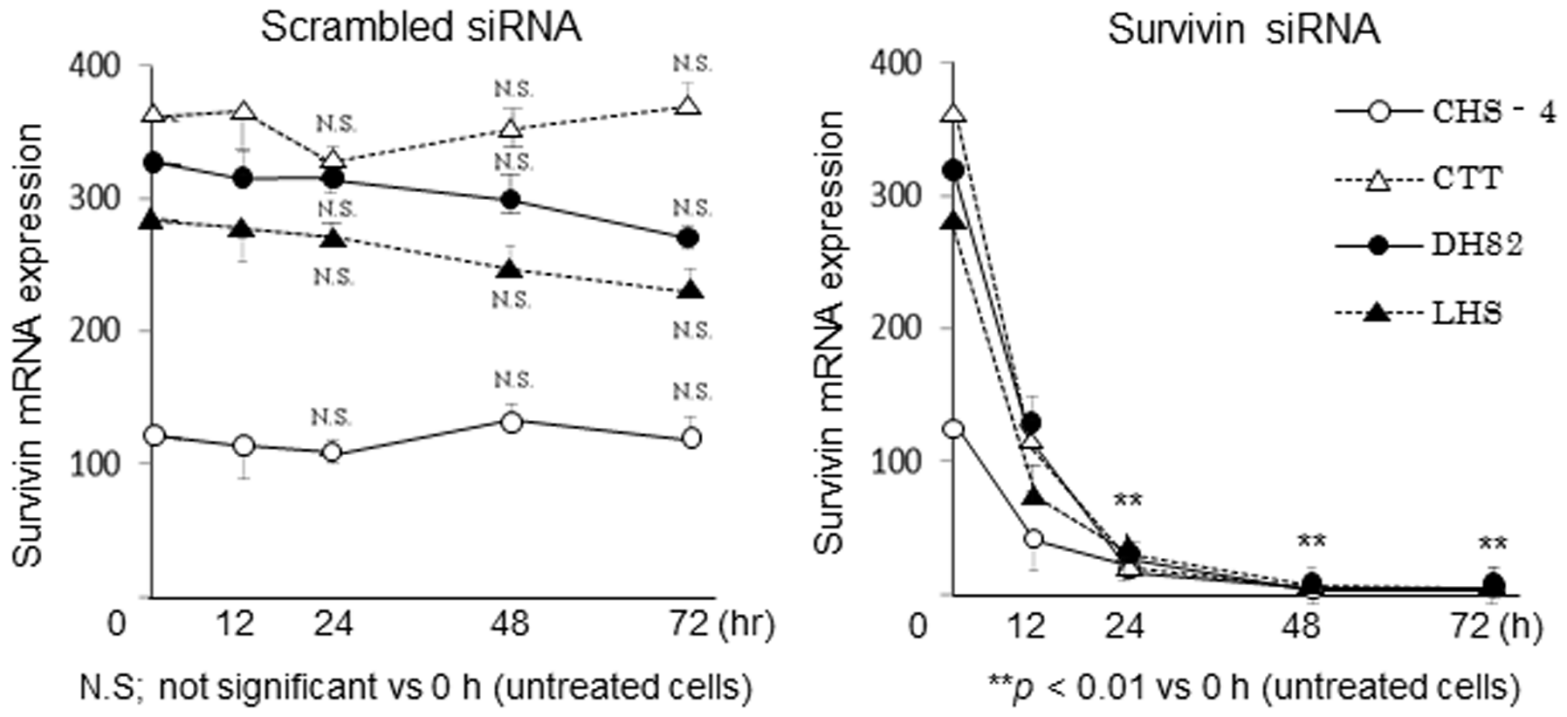
Expression of survivin mRNA in cell lines after transfection with siRNA. Expression levels of survivin mRNA in canine histiocytic sarcoma (CHS) cell lines were analyzed using qRT-PCR at 0, 12, 24, 48, and 72 h after transfection with siRNA, and 0 h point showed basal level of survivin mRNA in each CHS cell line before transfection. Expression levels of each gene were normalized to those of the same target gene in canine fibroblasts. Each bar represents the mean ± SE from 3 separate experiments. Data were statistically analyzed by one-way ANOVA followed by post-hoc test.

**Figure 3:**
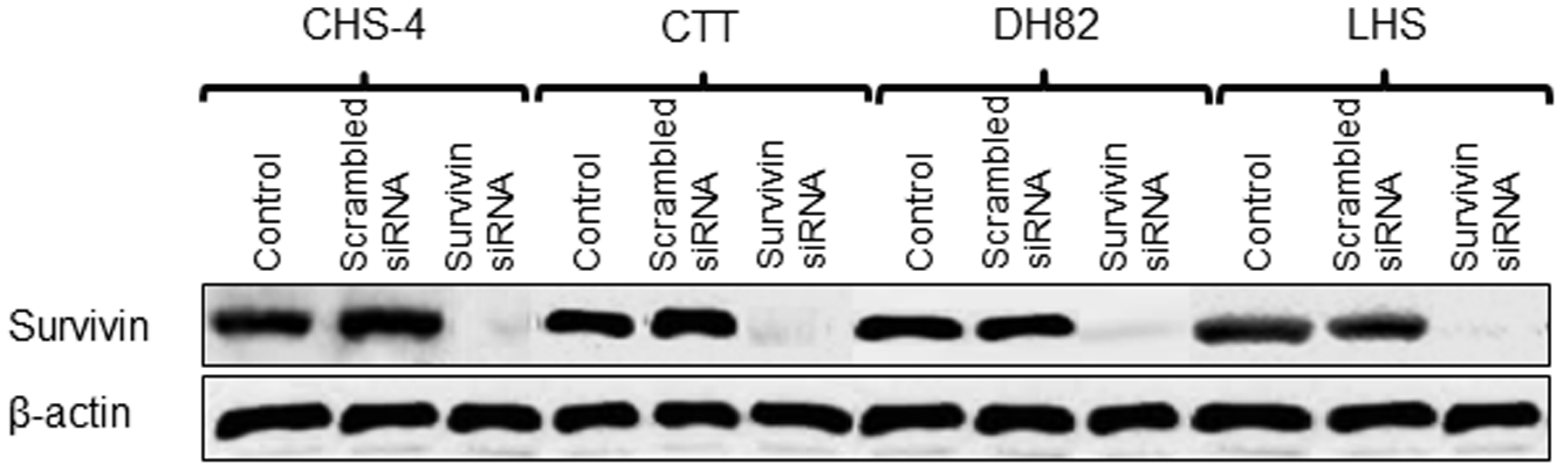
Expression of survivin protein in cell lines after transfection with siRNA. Survivin protein expression in CHS cell lines was evaluated by western blotting at 48 h after transfection with scrambled and survivin siRNA.

Basal information on the cell lines used in this study is shown in Table 2. To verify the transfection efficiency of the siRNA, survivin mRNA expression was measured at 0, 12, 24, 48, and 72 h after transfection (Fig. 2), and data at 0 h showed basal levels of survivin mRNA in each CHS cell line before transfection (Table 2). Survivin mRNA expression was significantly decreased by at least 90% in all CHS cell lines at 24-72 h after transfection with siRNA, while scrambled siRNA had only negligible effects on survivin expression. Western blotting analysis confirmed that survivin protein was downregulated in all CHS cell lines at 48 h after transfection with survivin siRNA (Fig. 3); scrambled siRNA transfection did not affect survivin protein expression in any cell line. All PCR products were identified as the target genes by sequence analysis.

**Table 2:** Information on cell lines used in this study.

			mRNA expression ^b)^			
Cell lines	Primary lesion	Doubling time (h) ^a)^	Survivin	MGMT	ABCB1	ABCC2
CHS-4	Synovium	75	124	36	28	24
CTT	Skin	46	360	86	76	69
DH82	Unknown	57	324	124	96	88
LHS	Lung	48	282	76	42	82
Fibroblast	Subcutis	60	1	1	1	1

a) Cells (1–2×104 cells/well) were seeded with DMEM supplemented with 10% FBS in 96-well plates, and each doubling time was researched by MTT assay.

b) Quantitative analysis using real-time PCR was perfomed. All expression levels in cell lines were normalized to those of the same mRNA in fibroblast.

MGMT: O(6)-methylguanine-DNA methyltransferase.

ABCB1: ATP-binding cassette transporter B1.

ABCC2: ATP-binding cassette transporter C2.

### Induction of apoptosis by survivin inhibition

**Figure 4:**
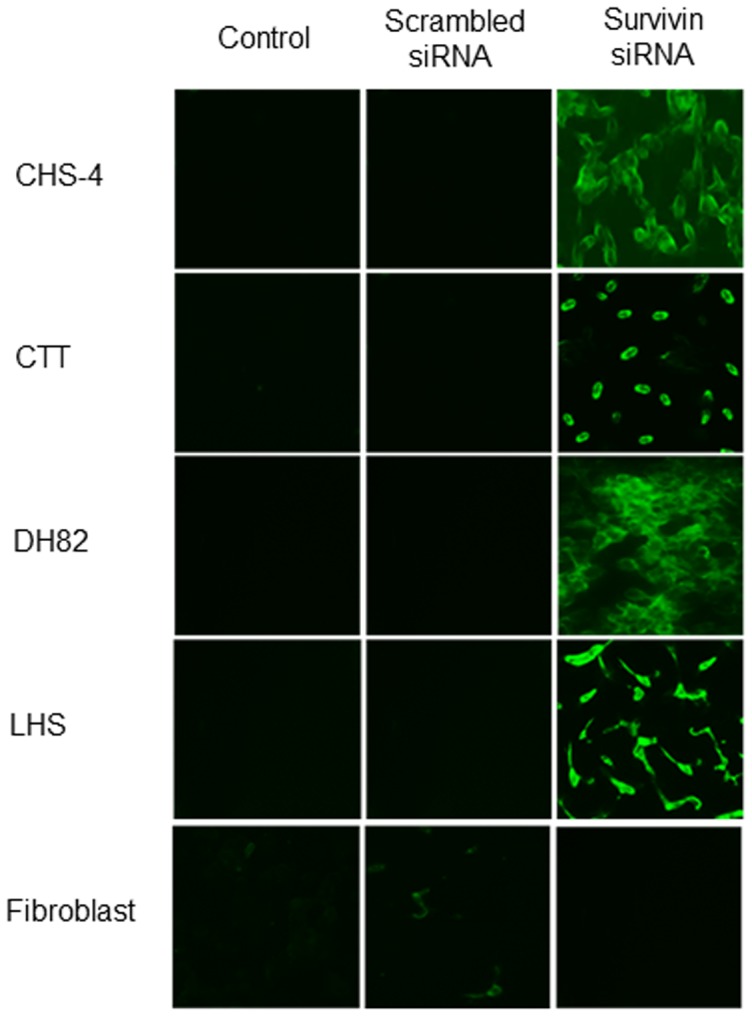
Influences of apoptosis after transfection with siRNA. Apoptotic cells in CHS cells and canine fibroblasts were evaluated using annexin V fluorescent staining at 48 h after transfection with scrambled and survivin siRNA (400 × magnification).

**Figure 5:**
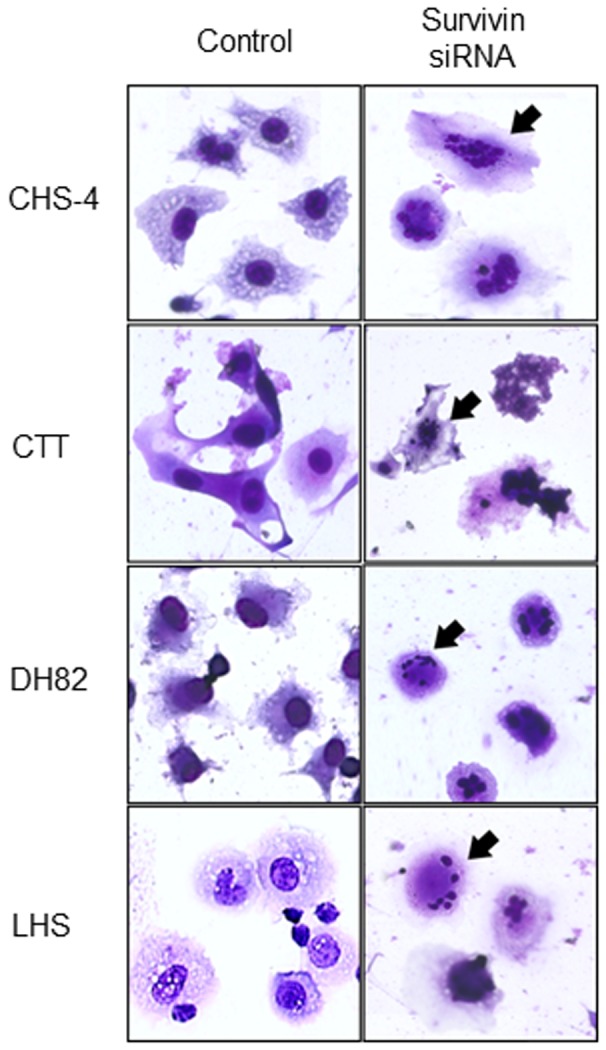
Imaging of the apoptotic cells after transfection with siRNA. Imaging of light microscope in CHS cell lines were performed by Wright-Giemsa staining at 48 h after transfection with survivin siRNA (1000 × magnification). Black arrows (?) showed nuclear fragmentation of CHS cells, which was observed at late-stage of the apoptotic process.

At 24 h after transfection with siRNA, annexin V staining was performed to detect apoptotic cells (Fig. 4). After transfection with survivin siRNA, apoptosis was observed in all CHS cells, while apoptosis was not observed in canine fibroblasts and CHS cells transfected with scrambled siRNA. In these apoptotic cells, fragmentation or swelling of cell nuclei was observed by Wright-Giemsa staining (Fig. 5).

### Influence of survivin knockdown on cell viability

**Figure 6:**
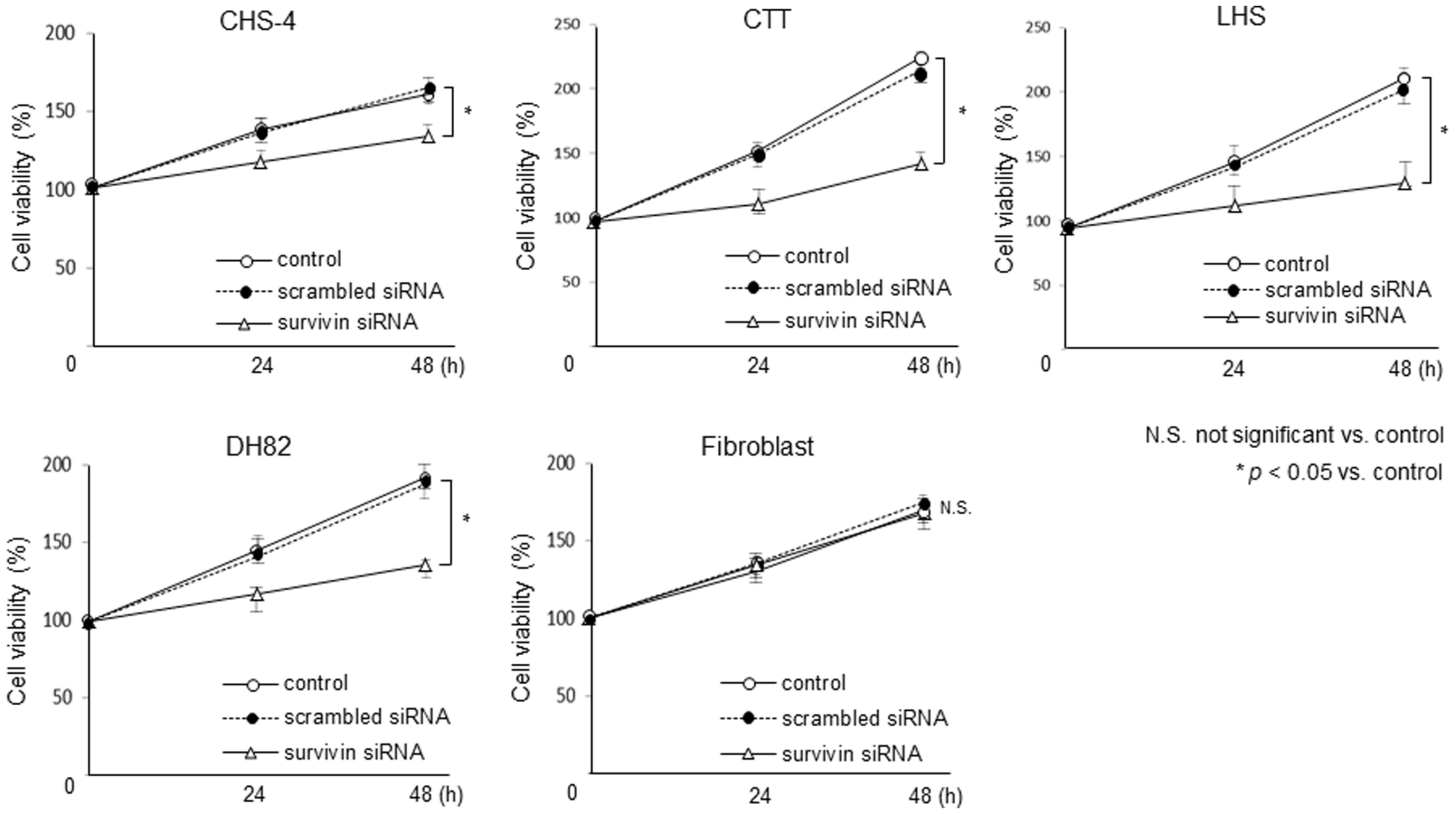
Influences of cell viability after transfection with siRNA. Cell viability was evaluated by methylthiazole tetrazolium (MTT) assay in CHS cell lines and canine fibroblasts at 24 or 48 h after transfection with scrambled and survivin siRNA. Each bar represents the mean ± SE from 3 separate experiments. *p<0.05 and not significant; N.S. vs. control cells (Post-hoc test).

At 24 and 48 h after transfection with siRNA, cell viability was evaluated (Fig. 6). After transfection with survivin siRNA, the viability of all CHS cells was significantly decreased as compared to that of the control. In contrast, canine fibroblasts and cells transfected with scrambled siRNA exhibited no changes in cell viability as compared to the control. Of the CHS cell lines, CTT and LHS cells exhibited the greatest difference in cell viability in response to survivin knockdown when compared to the other 2 CHS cell lines.

### Influence of survivin knockdown on CHS cell chemosensitivity

The sensitivities of CHS cells to CCNU and DOX after transfection with siRNA were evaluated (Table 3). After transfection with survivin siRNA, the IC50s of CCNU and DOX decreased in all cell lines. Additionally, the IC50 of CCNU was the lowest in DH82 cells (one-twelfth that before transfection), while the IC50 of DOX was the lowest in LHS cells (one-eighth that before transfection). In contrast, the IC50s of these drugs were not significantly affected by transfection with scrambled siRNA in any cell line (data not shown).

**Table 3:** 50% inhibitory concentrations of lomustine and doxorubicin in CHS cell lines.

	Lomustine (µM)		Doxorubicin (µM)	
Cell lines	Pre^ a)^	Post^ b)^	Pre^ a)^	Post^ b)^
CHS-4	18.2	3.2	2.4	0.8
CTT	96.6	24.6	12.6	6.6
DH82	86.4	7.2	8.6	1.8
LHS	42.6	18.6	18.4	2.4

a) Pre; No treatment.

b) Post; At 48 h after transfection with survivin siRNA.

**Figure 7:**
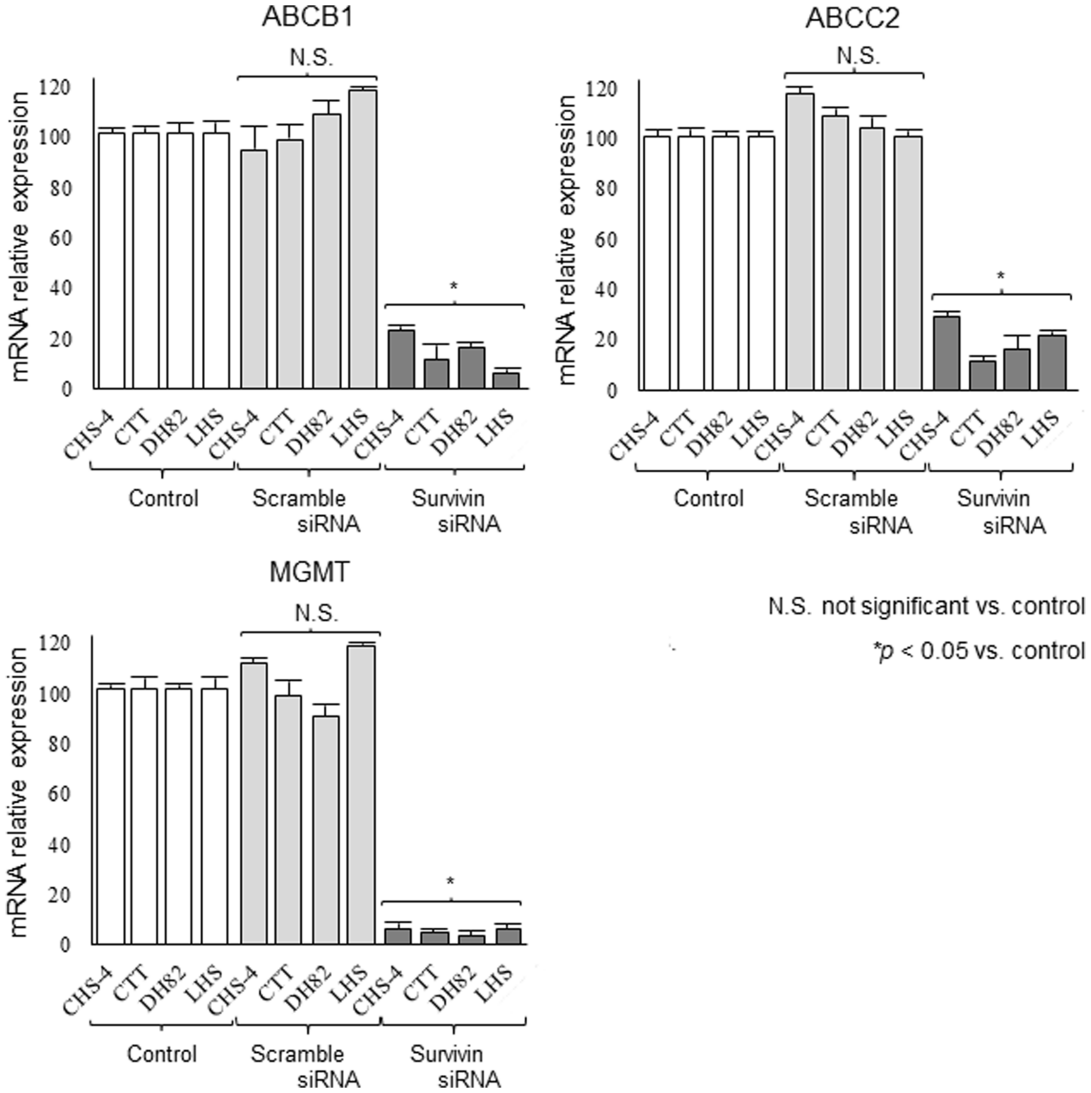
Expression of chemoresistance genes in cell lines after transfection with siRNA. The mRNA expression levels of chemoresistance genes, including ATP-binding cassette transporter B1 (ABCB1), ATP-binding cassette transporter C2 (ABCB2) and O (6)-methylguanine-DNA methyltransferase (MGMT), in CHS cells lines were evaluated at 48 h after transfection with scrambled or survivin siRNA. Expression levels of each gene were normalized to those of the same gene in untreated CHS cells (control) and are represented as the relative expression (% of control). Each bar represents the mean ± SE from 3 separate experiments. *p<0.05 and N.S. vs. control (Dunnett’s test).

The expression levels of ABCB1, ABCC2 and MGMT mRNAs were evaluated (Fig. 7). After transfection with survivin siRNA, the expression of these mRNAs were significantly decreased in all cell lines as compared to the untransfected control; scrambled siRNA transfection did not affect the expression of any of these targets. All PCR products were identified as the target genes by sequence analysis, and local sequence homology between survivin siRNA and PCR products, including ABCB1, ABCC2 and MGMT, were discontigous.

### Influence of survivin knockdown on protein expression and function of ABCB1

**Figure 8:**
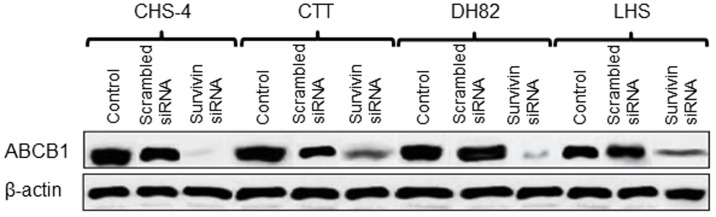
Expression of ABCB1 protein in cell lines after transfection with siRNA. ABCB1 protein expression in CHS cell lines was evaluated by western blotting at 48 h after transfection with scrambled or survivin siRNA.

**Figure 9:**
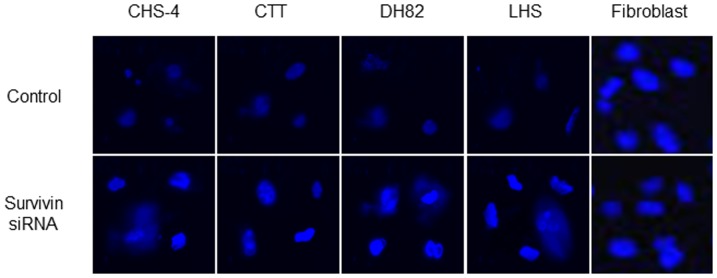
Influences of ABCB1 function in cell lines after transfection with siRNA. P-glycoprotein (ABCB1) pump activity was assessed by staining with Hoechst-33342 using fluorescence imaging in CHS cell lines and canine fibroblasts at 48 h after transfection with survivin siRNA (400× magnification).

At 48 h after transfection with survivin siRNA, expression of ABCB1 protein was downregulated in CHS cell lines as compared to control and scrambled siRNA-transfected cells (Fig. 8). In addition, survivin knock-down weakened the Hoechst efflux activity that Hoechst 33342 staining intensity increased in the nuclear and cell membrane of all CHS cell lines as compared to control and scrambled siRNA-transfected cells (Fig. 9 and data not shown), while this change was not observed in canine fibroblasts.

### Influence of survivin knockdown on phagocytic activity

**Figure 10:**
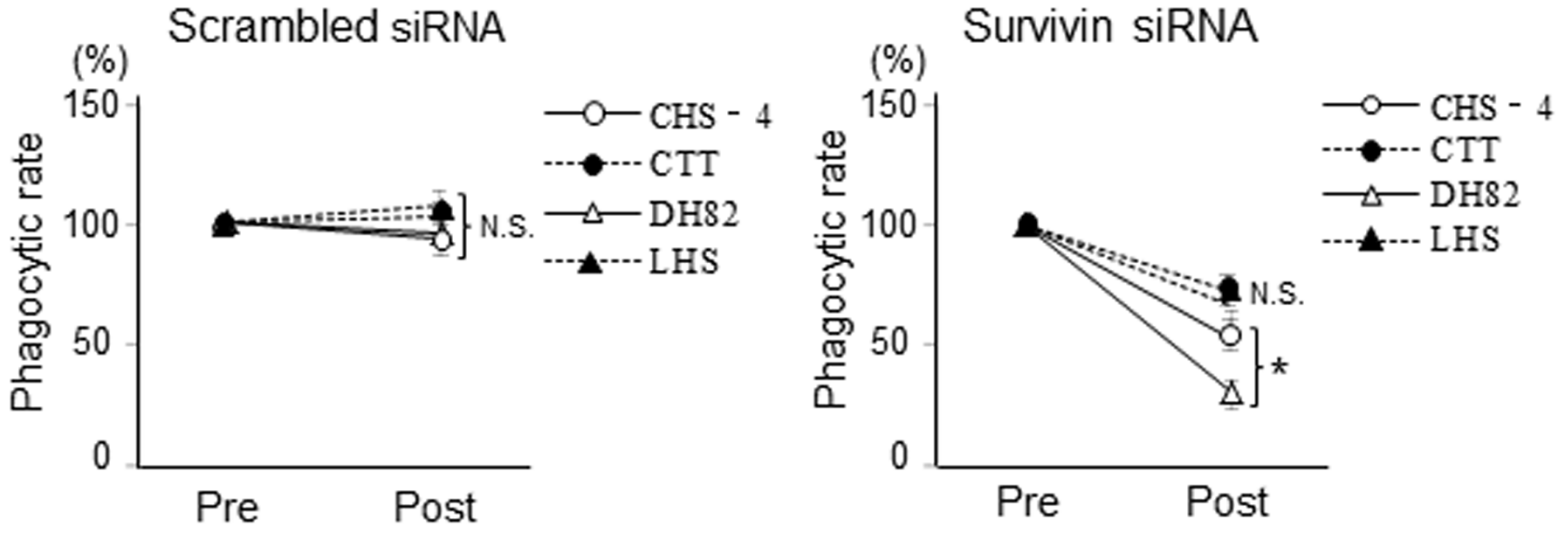
Influences of phagocytic activity after transfection with siRNA. Phagocytic rate of latex beads in CHS cell lines was evaluated at 48 h after transfected with scrambled and survivin siRNA as compared to that of untreated cells (pre). Total number of phagocytosed latex beads in 2,000 randomly selected, viable CHS cells was measured using an image analyzer. The relative phagocytic rate (% of pre) is shown. Each bar represents the mean ± SE from 3 separate experiments. *p<0.05 and N.S. pre (Dunnett’s test).

Phagocytic activity was evaluated by latex beads assay (Fig. 10). After transfection with survivin siRNA, total number of phagocytosed latex beads in 2 of the 4 CHS cell lines (CHS-4 and DH82) was significantly decreased as compared to control cells (median values; 58% and 36% of control cells, respectively). The total number of CTT and LHS also decreased as compared to control cells (median values; 74% and 69% of the control, respectively), however, these differences were not statistically significant. The phagocytic rates of all CHS cells transfected with scrambled siRNA were almost the same as that of the control.

## Discussion

In the present study, we investigated the effects of survivin knockdown in CHS cell lines in order to determine the potential efficacy of survivin-targeted therapy for the treatment of CHS. Several studies have shown that many anti-apoptotic genes and proteins, including Bcl-2 and IAP family members, are expressed at high levels in human and canine malignant tumors, including melanoma and osteosarcoma [13], [34]. Our studies showed that median expression levels of survivin mRNA in 4 CHS cell lines and clinical specimens collected from 30 HS dogs were significantly elevated compared to the expression levels of other Bcl-2 and IAP family members, including Bcl-2, Bcl-xL, mcl-1, cIAP-1, cIAP-2, and XIAP. Therefore, survivin may be specifically and highly expressed in CHS. Additionally, the presented study showed that the expression levels of anti-apoptotic genes, including Bcl-2, Bcl-xL, mcl-1, cIAP-1, cIAP-2, and XIAP, were not affected by knockdown of survivin by siRNA in 4 CHS cells lines. Therefore, our results demonstrated the specific effects of survivin, unaffected by changes in the expression of other anti-apoptotic genes.

Cell viability was significantly decreased in all CHS cell lines, but not normal canine fibroblasts, following transfection with survivin siRNA. Survivin protein is synthesized and expressed at high levels during the G2/M-phase of the cancer cell division cycle, effectively supporting the active cell growth process [11], [34], [35]. Therefore, inhibition of survivin could preferentially block cell division. In the present study, differences in the viabilities of 4 CHS cell lines were detected at 24 h after transfection with survivin siRNA, while other phenomena, including enhanced chemosensitivities and decreased phagocytic activity, were detected at 48 h after transfection. Thus, we suggested that inhibition of survivin might induce a preferential decrease in cell viability by apoptosis and delay the progressive pathological process of CHS cells, which have comparatively high cell growth potential in different types of CHS cells.

After transfection with survivin siRNA, the sensitivities of CHS cells to CCNU and DOX were significantly increased as compared to that of control cells. In addition, the results presented that survivin inhibition might change the expression patterns of multiple chemoresistance genes, including ABCB1, ABCC2 and MGMT, against all CHS cell lines. Some research groups have reported that inhibition of survivin may prevent acquisition of chemoresistance by anti-apoptotic mechanisms or enhancement of cytological telomerase activity, which prolongs the apoptotic process [10], [11]. Interestingly, our data supported the hypothesis that survivin inhibition induced downregulation of ABCB1 protein in CHS cell lines, resulting in inhibition of the P-glycoprotein pump. Some groups have also suggested that survivin-mediated transcription is associated with P-glycoprotein/MDR1 overexpression in human breast cancer cells [36], [37]. Other groups have demonstrated that cell transfected with the survivin gene may exhibit enhanced activity of specificity protein 1 as a transcription factor [38], and overexpression of survivin may affect NF-κB activity in cancer cells via feedback control [39], [40]. Activated specificity protein 1 and NF-κB induce the production of chemoresistance genes and proteins, including P-glycoprotein and ABC transporter [41]–[44]. We suggested that survivin inhibition might weaken expression and function of multiple chemoresistance factors through influencing these transcription factors. In the present study, western blotting analysis was also performed using antibodies targeting ABCC2 and MGMT for other species because we could not establish these antibodies for targeting canine-specific proteins. However, it was unable to identify cross-reactivity with ABCC2 and MGMT antibodies (data not shown). Further investigations, including protein expression and functional assessment of ABCC2 and MGMT based on survivin inhibition, are required for validation of this hypothesis.

After transfection with survivin siRNA, phagocytic activity was decreased in all CHS cell lines, and significant differences were observed in 2 of the 4 CHS cells. The ralationship between survivin expression and phagocytic activity against cancer cells has not been well studied. Several research groups have suggested that some physical activities in human cancer cells, including cell migration, invasion, and metastatic potential, are correlated with survivin expression [8], [44]–[46], and one group have demonstrated that upregulation of survivin enhances these activities in cancer cell lines, while downregulation of survivin blocks these activities [47]. These weakened phagocytic activity may be induced secondarily by changes in basal physical activities, such as invasive potential and motor activities, which CHS cells exhibit naturally, or variations in genetic pathways downstream of survivin [8], [35], [46]. Thus, the weakened phagocytic activity resulting from survivin knockdown in CHS cell lines may be the indirect result of survivin inhibition rather than a direct consequence of survivin inhibition. However, not all cell lines responded to knockdown of survivin to the same extent; CTT and LHS cells had low phagocytic activity before survivin knockdown compared to other cell lines. Therefore, further studies are required to determine the mechanisms through which survivin affects phagocytic activity in CHS cells.

Survivin target therapy is currently expected to be a potential effective therapy with low side effects for several malignant neoplasms, and clinical trials for human cancers are underway using survivin inhibitors. One such inhibitor is YM155, a small-molecule suppressor of survivin activity, and phase II clinical trials are currently underway YM155 in human cancer patients [48], [49]. Another survivin inhibitor, EZN-3042, is currently being investigated in phase I clinical trials in human patients [13], [50]. Hemophagocytic histiocytic sarcoma originating from macrophages has fundamentally more aggressive biological behaviors and poorer prognoses as compared to CHS with DC origins, and no effective treatments for this tumor type have yet been developed. In this study, we demonstrated that knockdown of survivin using siRNA induced changes in cell appearance and cell viability in all CHS cell lines tested, which were established from several different sites of primary lesions; moreover, knockdown of survivin also affected DH82 cells, which originated from macrophages. In contrast, normal canine fibroblasts were unaffected by inhibition of survivin expression. Therefore, these data suggested that survivin inhibitors, including YM155 and EZN-3042, have the potential to be efficacious against CHS. However, further studies using several CHS cell lines originating from macrophages are required to verify whether survivin inhibition may be effective for all types of CHS, regardless of differential cell origins.

In conclusion, the present study demonstrated that inhibition of survivin expression decreased cell viability through the induction of apoptosis, enhancement of chemosensitivity, and weakening of phagocytic activities in CHS cell lines. These findings suggested that survivin may partially support cell survival and maintain aggressive biological behaviors in CHS. Therefore, survivin may be an effective target for novel therapeutics or combination therapies with conventional anticancer drugs for CHS. Further studies are required to fully elucidate the antitumor effects of survivin inhibition in CHS.
